# Editorial: Novel Approaches to Teaching Scientific Thinking: Psychological Perspectives

**DOI:** 10.3389/fpsyg.2017.00820

**Published:** 2017-05-18

**Authors:** Rodney M. Schmaltz, Scott O. Lilienfeld

**Affiliations:** ^1^Department of Psychology, MacEwan UniversityEdmonton, AB, Canada; ^2^Department of Psychology, Emory UniversityAtlanta, GA, United States; ^3^School of Psychological Sciences, University of MelbourneMelbourne, Australia

**Keywords:** scientific thinking, misconceptions, cognitive biases, science teaching, creationism, dualism, signal detection, heavy metal music

Traditionally, the educational field has adhered to a “knowledge deficit” model of science learning in which the principal problem confronting students is a dearth of accurate knowledge (Bak, 2001). Nevertheless, filling up pupils' minds with facts and figures, although necessary, is rarely sufficient as an educational goal. Even with an adequate knowledge base, many students are incapable of evaluating assertions with a discerning and appropriately critical eye. Along with this recognition is an awareness that science is more than a body of knowledge; it is an epistemic *approach* to evidence (Sagan, 1995) that emphasizes error-reduction.

Indeed, scholars have espoused the need to promote scientific thinking for many years, even decades (e.g., Mackay, 1869; Gardner, 1957; Randi, 1982; Shermer, 2011). Often, historical treatises on the requirement for scientific skepticism begin with the notion that now, more than ever, we need to teach our students how think scientifically about claims in everyday life. Although it sounds cliched to say it again, the need to promote scientific thinking skills may indeed be needed now more than ever. The growing deemphasis on the importance of factual information, as well as the understandable difficulty many students encounter in distinguishing print, online, and media information from misinformation renders this Research Topic particularly timely.

Scientific thinking—the ability to generate, test, and evaluate claims in ways that minimize our inherent propensities toward bias (e.g., Koerber et al., 2015)—is crucially important for our students, who are continually exposed to nearly limitless information and misinformation online. In today's world, even legitimate news organizations at times promote invalid and misleading information. It can be exceedingly challenging for students, and even their instructors, to distinguish wheat from chaff and to accurately determine the validity of claims. This Research Topic in Frontiers in Educational Psychology focuses on strategies to help instructors promote sound scientific thinking. Even after extensive training in science at a postsecondary level, many pseudoscientific beliefs may persist (e.g., Winer et al., 2002). Hence, we may need to explore novel approaches to dispelling such beliefs in students. The articles presented here provide a wide range of approaches to promoting scientific thinking, and cover a range of topics from the misuse of psychological terms to user-friendly demonstrations in neuroscience.

A lack of understanding of the nature of science bears significant real world implications. For example, 40% of Americans do not believe there is a scientific consensus on climate change (National Science Board, Science and Engineering Indicators, 2016^1^), and the American president (as of the time of this writing), has endorsed such unsupported notions such as the debunked assertion that vaccines cause harm, or that global warning trends are a “hoax” (2016; Berezow and Campbell, 2017). In fairness to him, the embrace of dubious scientific claims, including those in psychology and allied fields, is clearly bipartisan (Duarte et al., 2015).

The papers in this issue adopt a broad and diverse approach to teaching scientific thinking. Lilienfeld et al. discuss 50 psychological and psychiatric terms that are inaccurate, commonly misused, or both. They discuss why these terms are often used incorrectly, and provide students and instructors alike with strategies to correct misconceptions of the terms, along with recommendations for preferable terms.

Matute et al. demonstrate the role of the illusion of causality in fostering continued belief in pseudoscience and misinformation. An overview of the innovative experiments in the Matate lab show that an understanding of the illusion of causality can promote of scientific thinking.

Hamilton and Hamilton explore how illusions that demonstrate key concepts in neuroscience can be profitably applied to philosophical arguments. In this regard, the authors place a particular emphasis on mind-body dualism, which is a deeply entrenched assumption among many beginning students.

The promotion of scientific thinking may be a valuable window into the discussion of controversial topics. For example, Honey describes the value of bringing supernatural views into the classroom. Specifically, she argues that if students are not exposed to the logical flaws of pseudoscientific or otherwise nonscientific views, they may continue to see supernatural perspectives, such as creationism, as viable alternatives to science. Schmaltz similarly proposes that controversial examples found in popular culture, such as the harm supposedly caused by listening to heavy metal music, can provide engaging examples to help students think like scientists.

Anderson discusses how pseudoscientific examples can be used to help students understand the value of signal detection theory. By incorporating engaging examples of pareidolia and psychic detectives, Anderson demonstrates how signal detection theory can frame how people make decisions regarding the accuracy of a claim.

For students, and the public at large, the ability to think like a scientist helps inform important decisions ranging from global issues, such as anthropogenic climate change, to personal issues, such as health choices (e.g., vaccine safety and dubious alternative medicine claims). This Research Topic offers readers with a wide range of valuable approaches to promoting scientific thinking. Ensuring that students are equipped with sound scientific thinking skills is no easy task, as people tend to trust their intuitions and are largely unaware of the biases that influence their decision making (Pronin et al., 2002; Lilienfeld et al., 2012). The approaches discussed in this Research Topic provide educators with a sampling of the tools necessary to safeguard students against the seductive appeal of pseudoscientific claims. With these tools, students should hopefully be better prepared to successfully sift through the reams of information—and misinformation—with which they are bombarded on a daily basis.

## Author contributions

SL is the lead author on this manuscript. Both authors have contributed to the writing of this editorial.

### Conflict of interest statement

The authors declare that the research was conducted in the absence of any commercial or financial relationships that could be construed as a potential conflict of interest.

## Footnotes

^1^National Science Board, Science and Engineering Indicators (2016). Arlington, VA: National Science Foundation (NSB-2016-1).
